# Supplementary material for: Reference Intervals for Blood Biomarkers in Farmed Atlantic Salmon, Coho Salmon and Rainbow Trout in Chile: Promoting a Preventive Approach in Aquamedicine
Source: Biology (Basel). 2022 Jul 18;11(7):1066. doi: 10.3390/biology11071066 (PMC9312075; doi:10.3390/biology11071066)
Supplement: Supplementary file 1 [file biology-11-01066-s001.zip › biology-1770680-supplementary.pdf]

Supplementary materials Figure S1–S18. Supplementary analyses and figures

Marco Rozas-Serri*, Rodolfo Correa, Romina Walker-Vergara, Darling Coñuecar, Soraya Barrientos, Camila Leiva, Ricardo Ildefonso, Carolina Senn, Andrea Peña

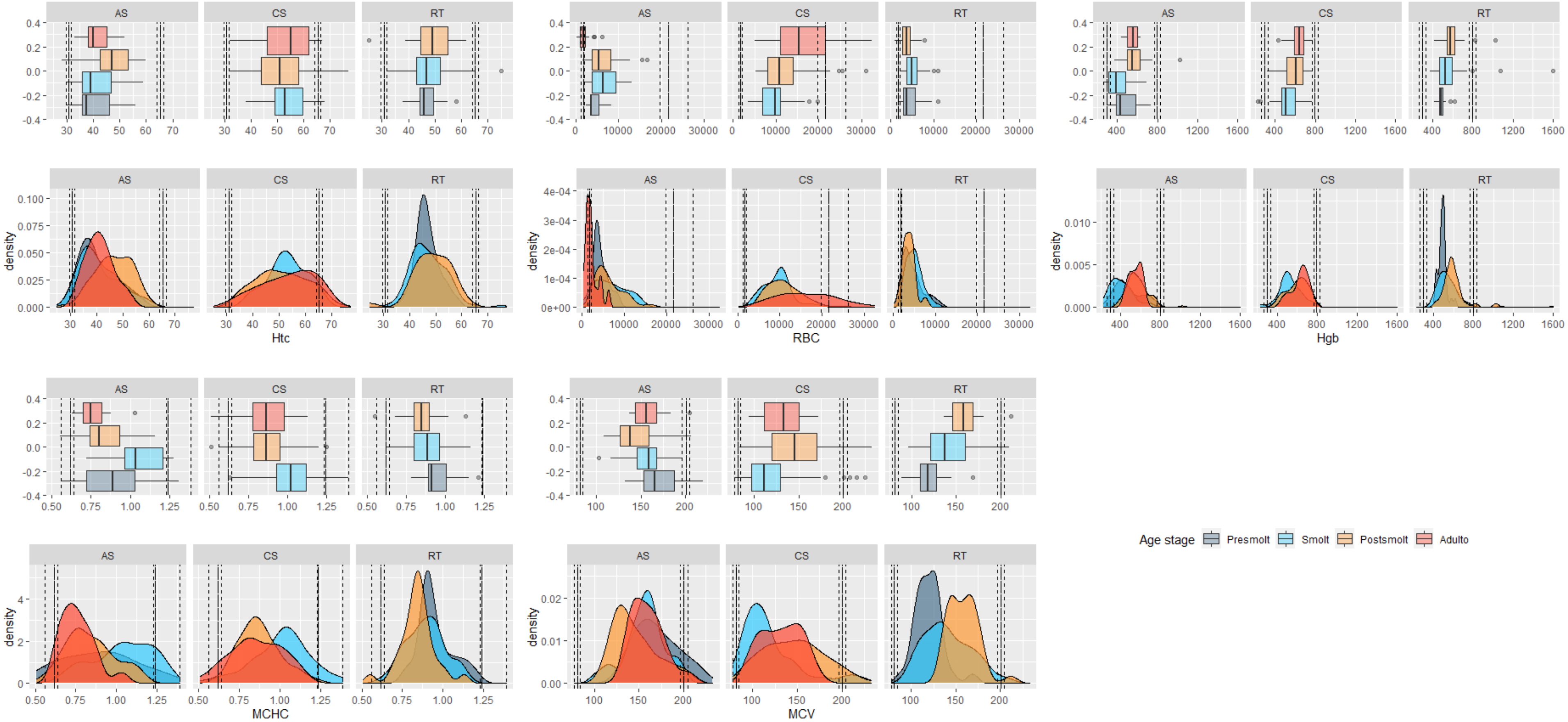

Figure S1. Histograms represent the count of erythrogram biomarker observations and density plots show the distribution of data by estimating kernel density among salmonid species and age ranges. Box plots show the overall IRs (black lines) for erythrogram biomarkers. Dashed black lines show CIs for both the maximum and minimum range of RIs. AS: Atlantic salmon; CS: Coho salmon; RT: Rainbow trout.

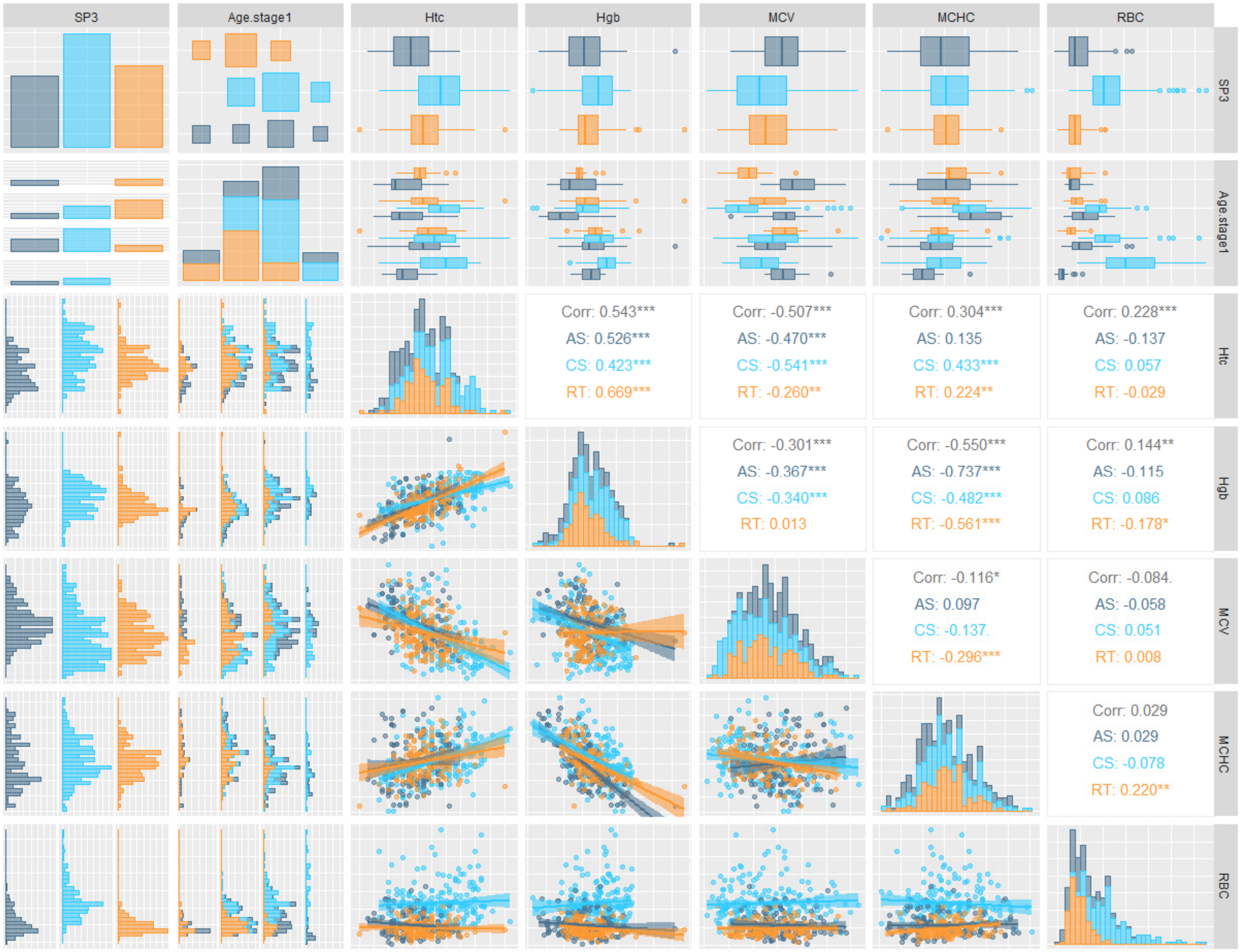

Figure S2. Correlogram between the different erythrogram biomarkers according to salmonid species. Pearson correlation coefficient (r) and p-value (p) between Hct, Hgb, RBC, MCV, MHCH in Atlantic salmon (AS), coho salmon (CS) and rainbow trout (RT) (*p < 0.05, **p < 0.01, ***p < 0.001). SP: Specie.

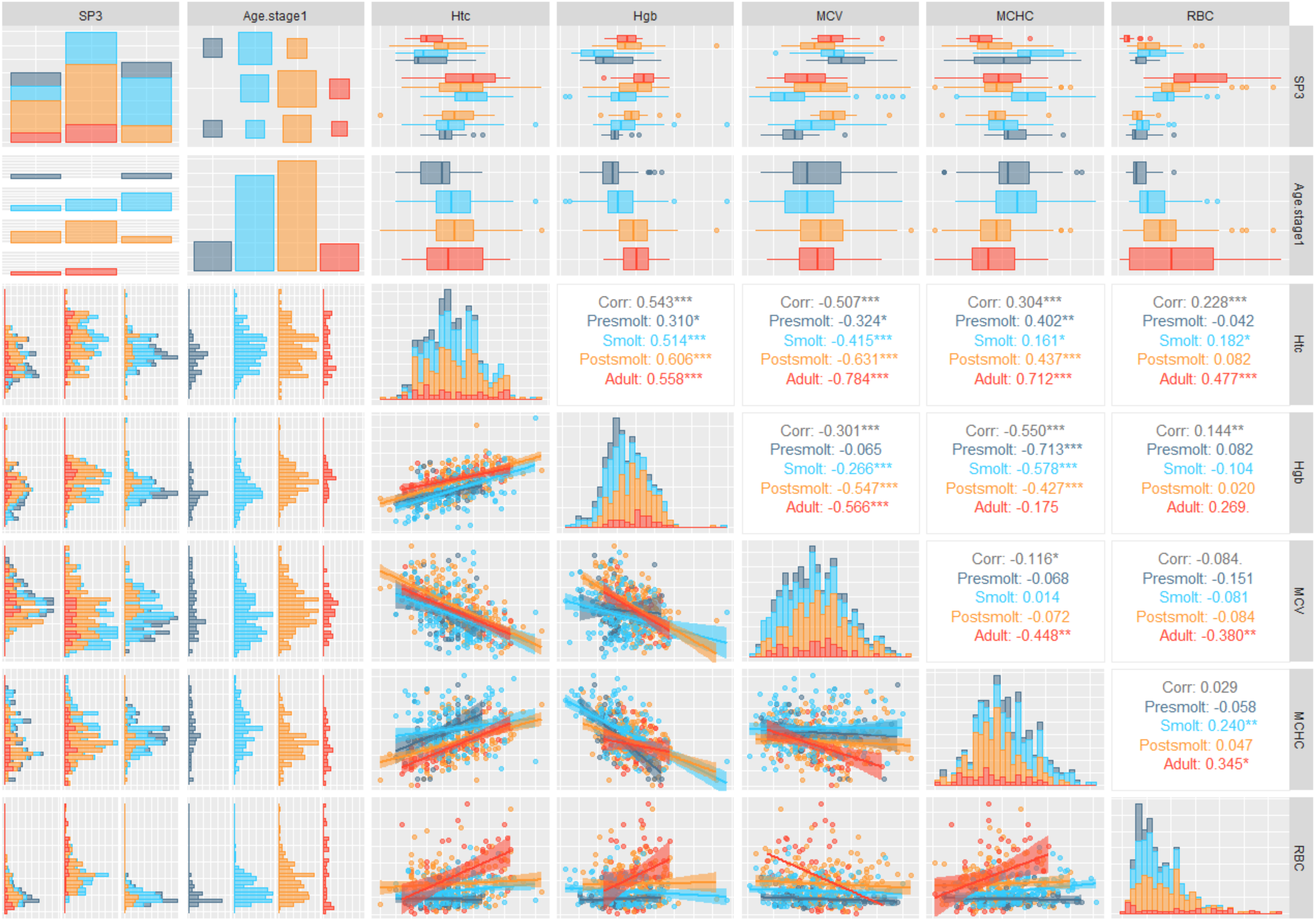

Figure S3. Correlogram between the different erythrogram biomarkers according to age ranges. Pearson correlation coefficient (r) and p-value (p) between Hct, Hgb, RBC, MCV, MHCH in presmolt, smolt, postsmolt and adult from Atlantic salmon (AS), coho salmon (CS) and rainbow trout (RT) (*p < 0.05, **p < 0.01, ***p < 0.001). SP: Specie.

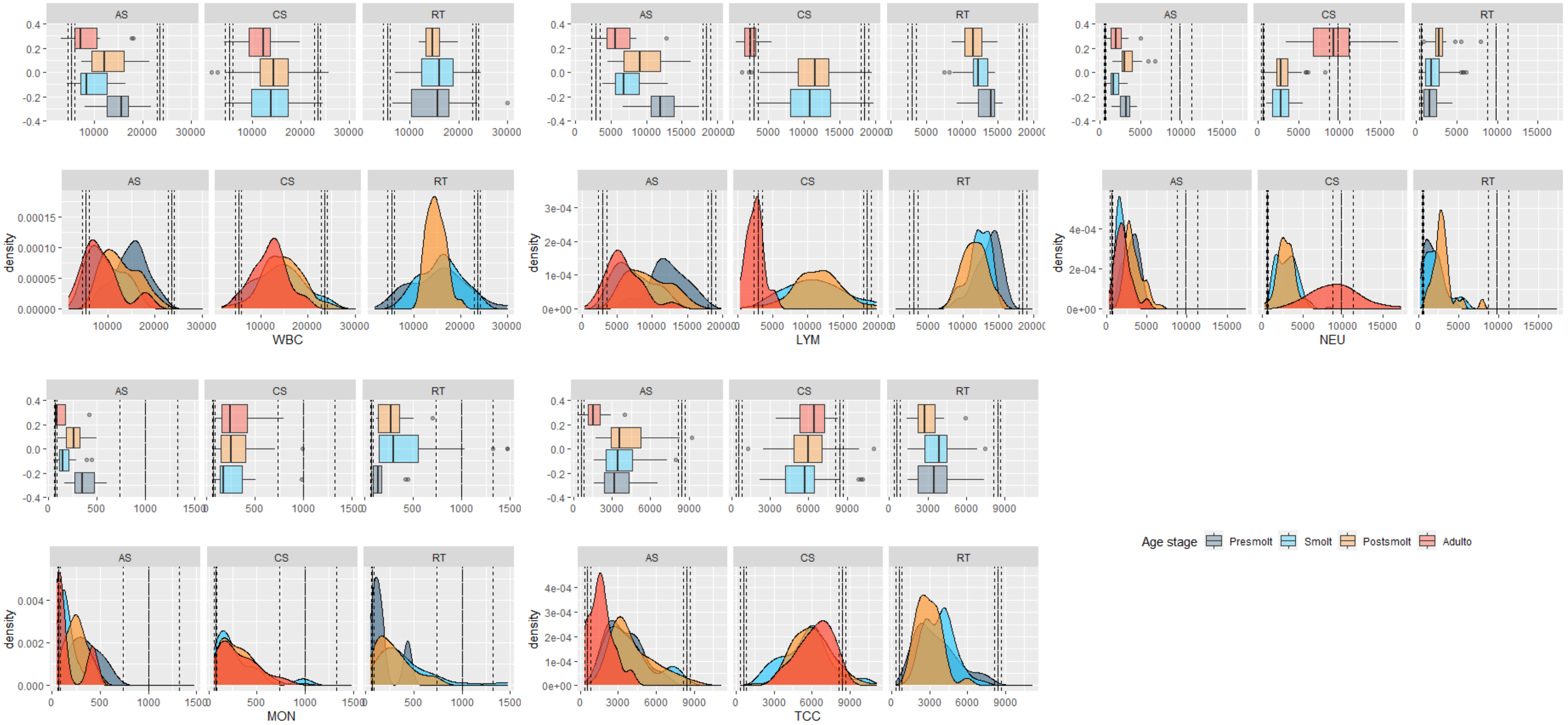

Figure S4. Histograms represent the count of leukogram biomarker observations and density plots show the distribution of data by estimating kernel density among salmonid species and age ranges. Box plots show the overall IRs (black lines) for leukogram biomarkers. Dashed black lines show CIs for both the maximum and minimum range of RIs. AS: Atlantic salmon; CS: Coho salmon; RT: Rainbow trout.

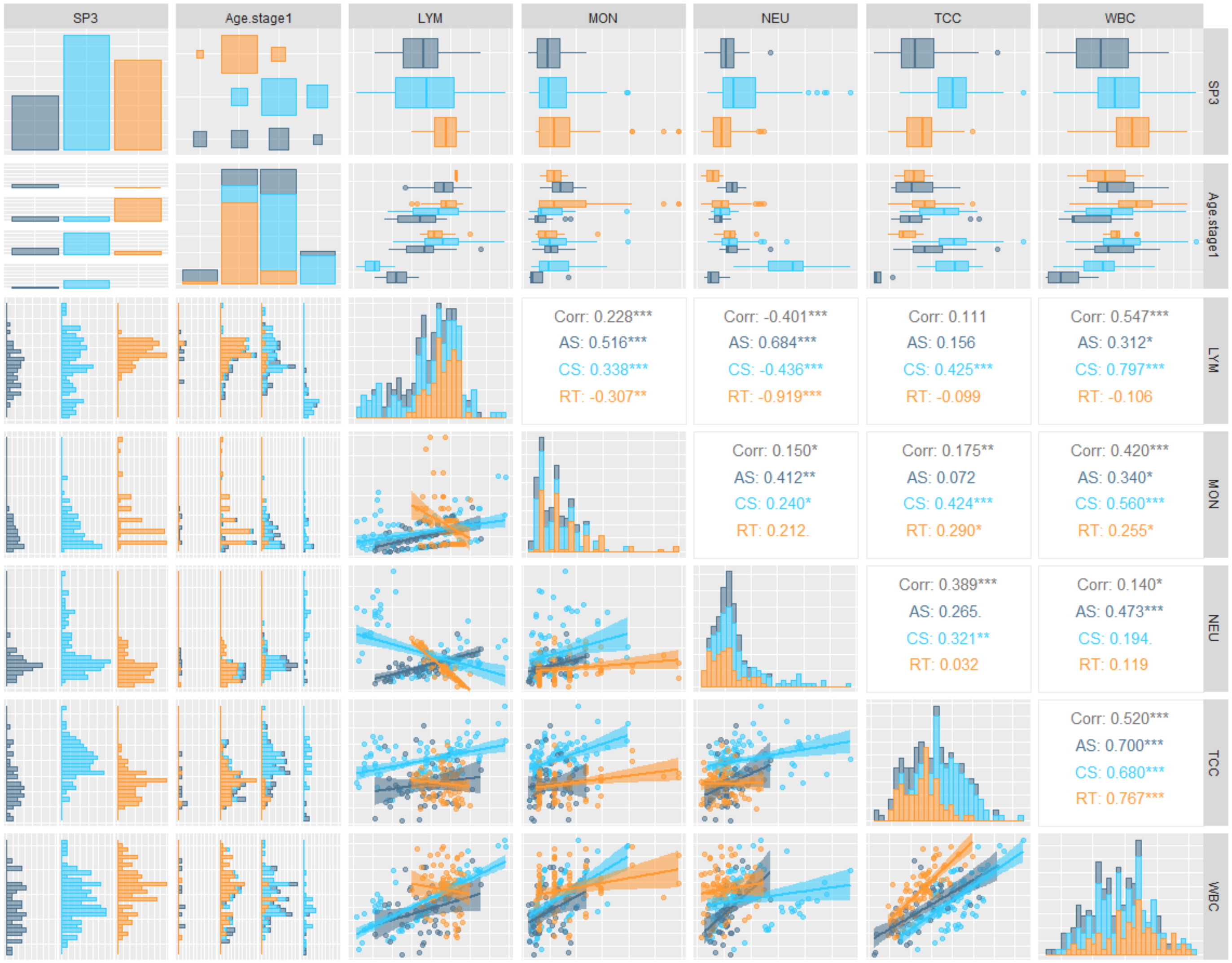

Figure S5. Correlogram between the different leukogram biomarkers according to salmonid species. Pearson correlation coefficient (r) and p-value (p) between WBC, LYM, NEU, MON, and TCC in Atlantic salmon (AS), coho salmon (CS) and rainbow trout (RT) (*p < 0.05, **p < 0.01, ***p < 0.001). SP: Specie.

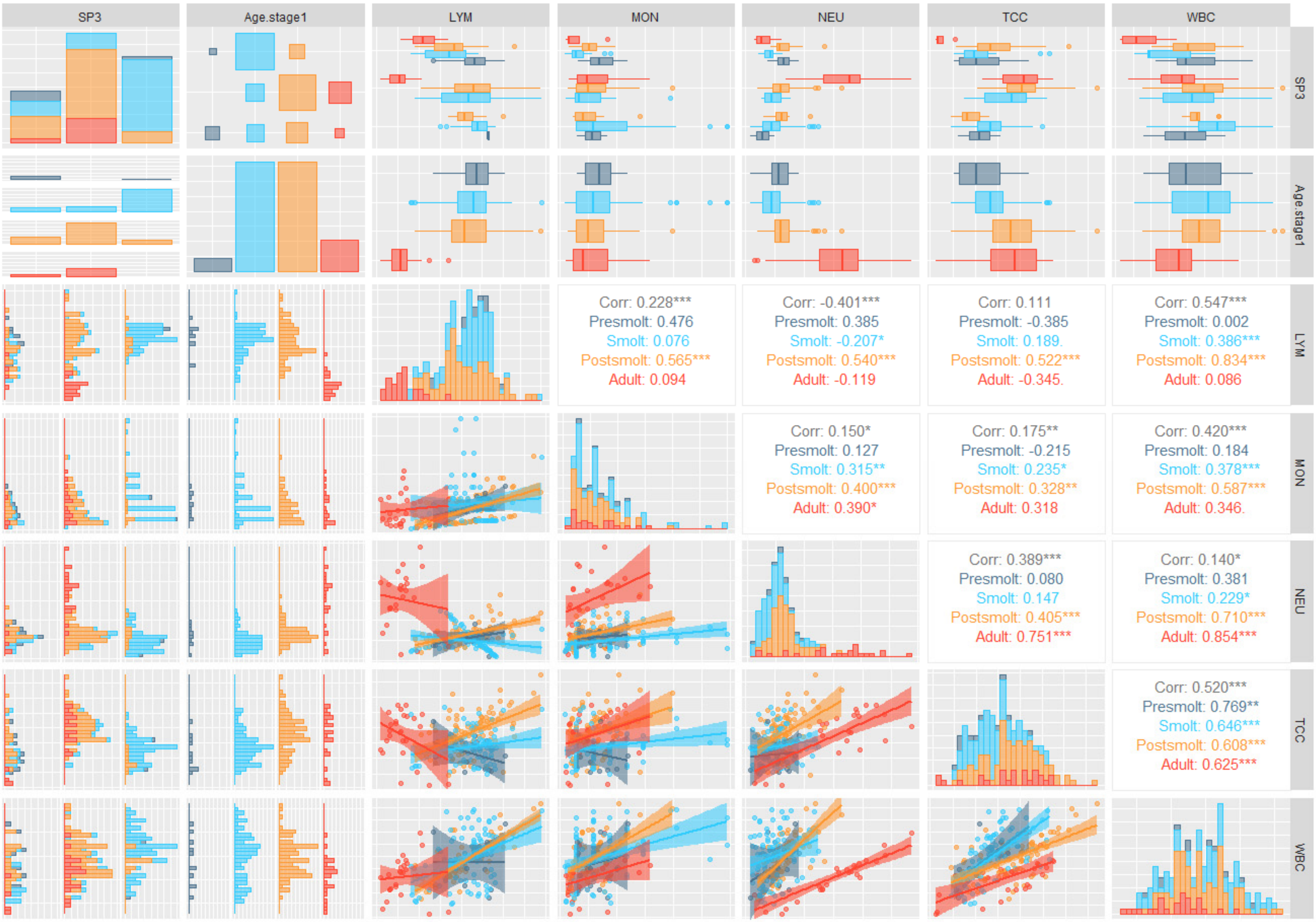

Figure S6. Correlogram between the different leukogram biomarkers according to age ranges. Pearson correlation coefficient (r) and p-value (p) between WBC, LYM, NEU, MON, and TCC in presmolt, smolt, postsmolt and adult from Atlantic salmon (AS), coho salmon (CS) and rainbow trout (RT) (*p < 0.05, **p < 0.01, ***p < 0.001). SP: Specie.

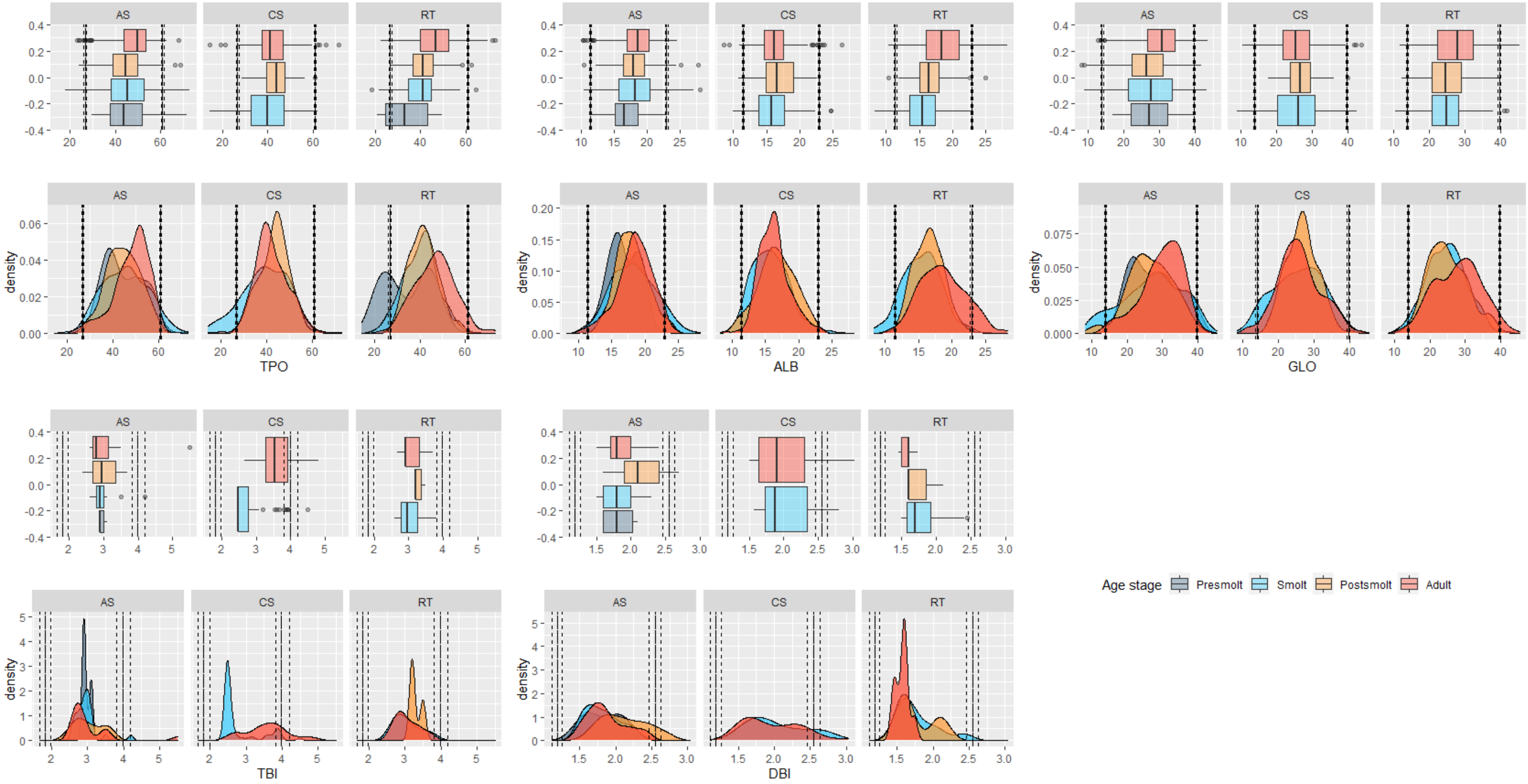

AS
CS
RT
density
TPO
ALB
GLO
TBI
DBI
Age stage
Presmolt
Smolt
Postsmolt
Adult

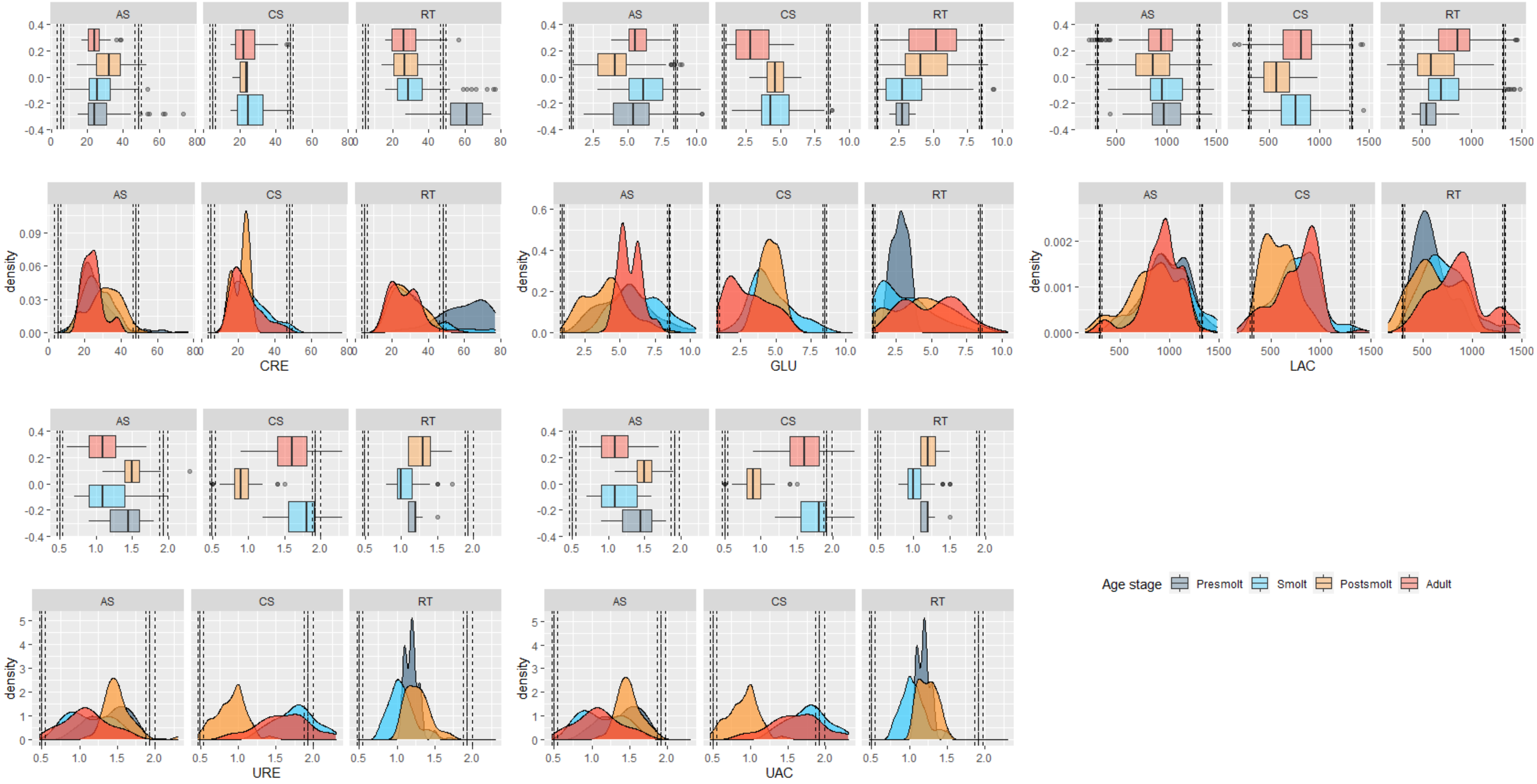

AS
CS
RT
density
CRE
GLU
LAC
URE
UAC
Age stage
Presmolt
Smolt
Postsmolt
Adult

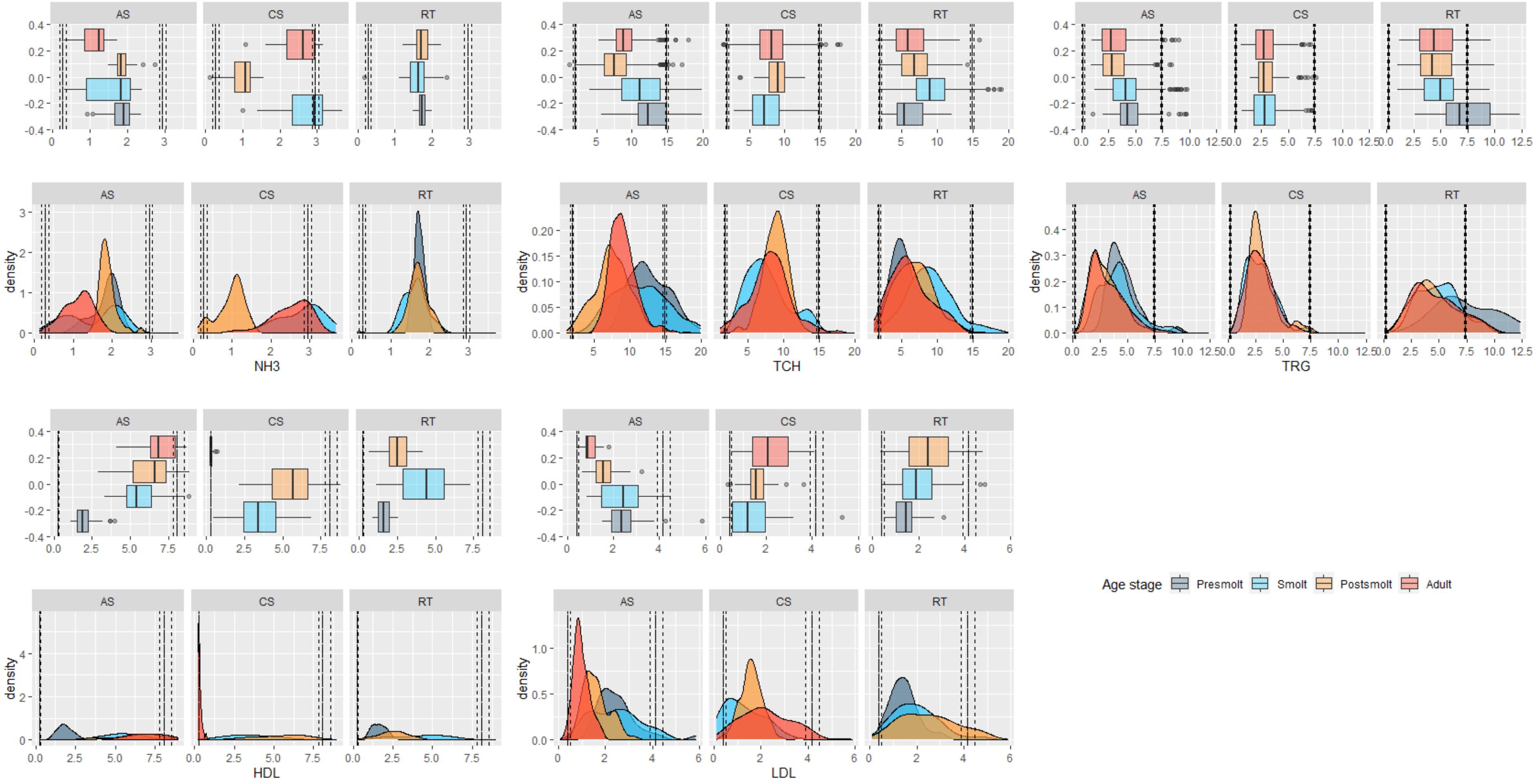

Figure S7. Histograms represent the count of plasma substrates biomarkers observations and density plots show the distribution of data by estimating kernel density among salmonid species and age ranges. Box plots show the overall IRs (black lines) for plasma substrates biomarkers. Dashed black lines show CIs for both the maximum and minimum range of RIs. AS: Atlantic salmon; CS: Coho salmon; RT: Rainbow trout.

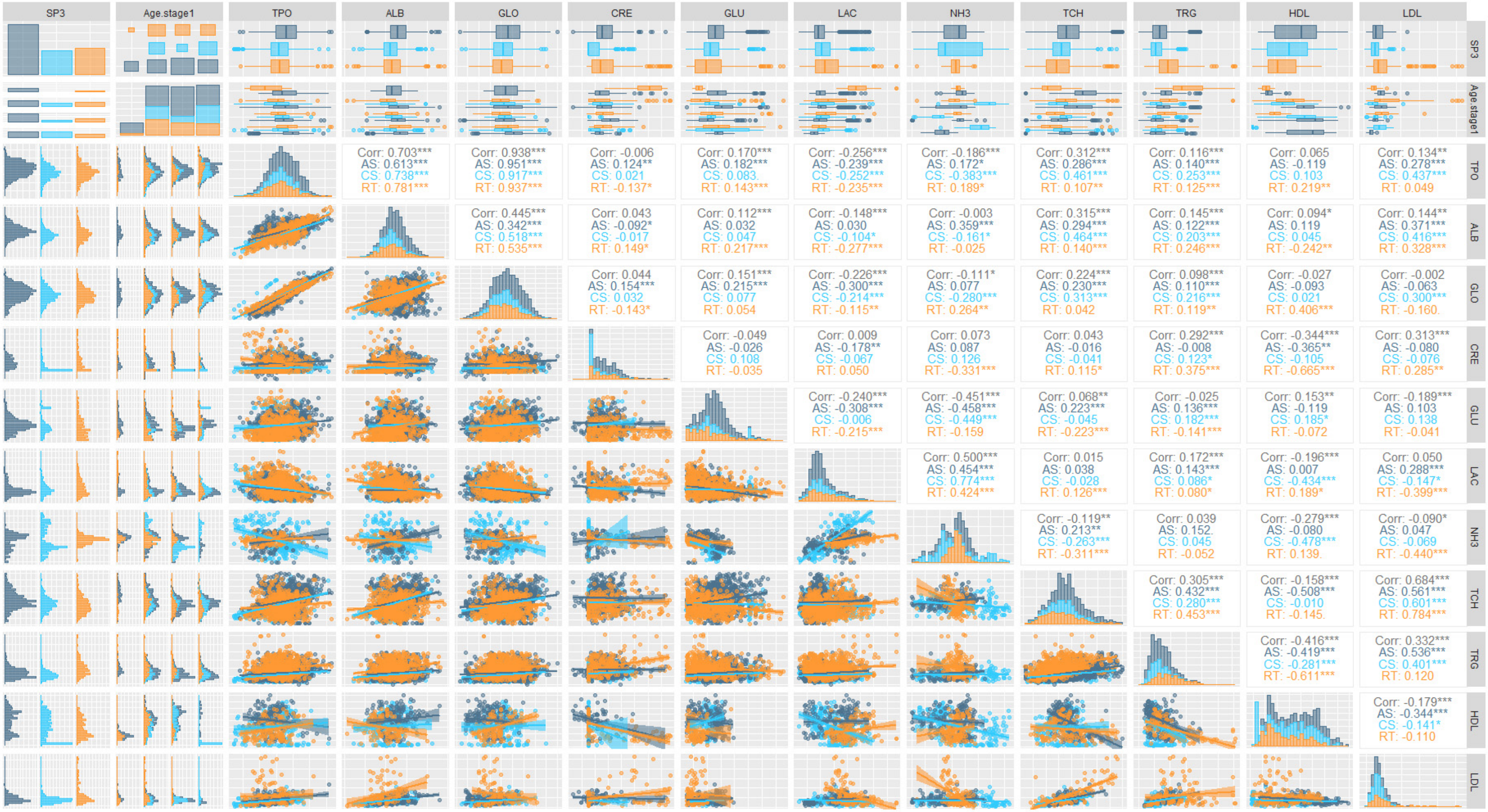

Figure S8. Correlogram between the different plasma substrates biomarkers according to salmonid species. Pearson correlation coefficient (r) and p-value (p) between TPO, ALB, GLO, TBI, DBI, CRE, GLU, LAC, NH3, URE, AUC, TCH, TRG, HDL, LDL, and COR in Atlantic salmon (AS), coho salmon (CS) and rainbow trout (RT) (*p < 0.05, **p < 0.01, ***p < 0.001). SP: Specie.

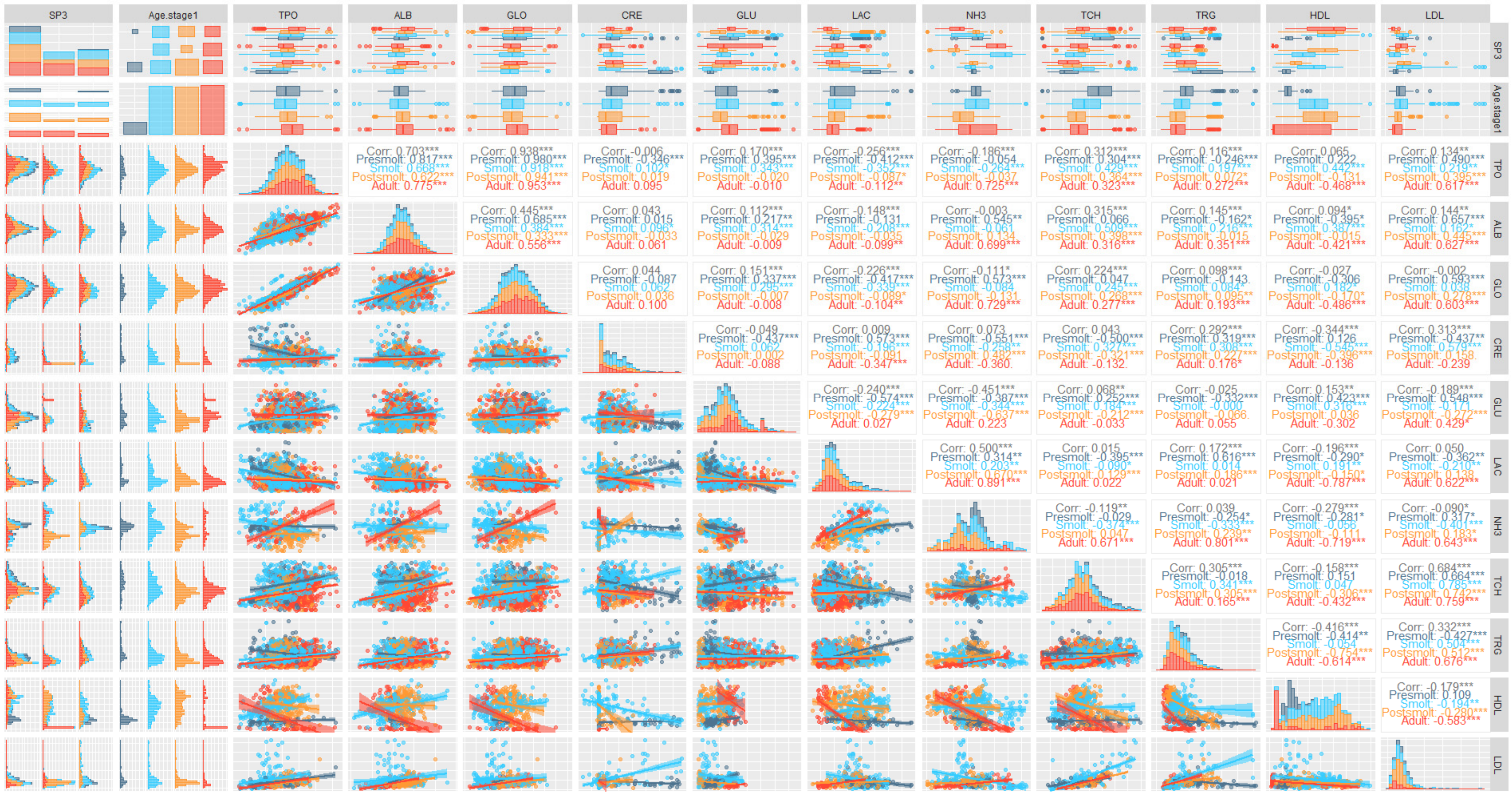

Figure S9. Correlogram between the different plasma substrates biomarkers according to age ranges. Pearson correlation coefficient (r) and p-value (p) between TPO, ALB, GLO, TBI, DBI, CRE, GLU, LAC, NH3, URE, AUC, TCH, TRG, HDL, LDL, and COR in presmolt, smolt, postsmolt and adult from Atlantic salmon (AS), coho salmon (CS) and rainbow trout (RT) (*p < 0.05, **p < 0.01, ***p < 0.001). SP: Specie.

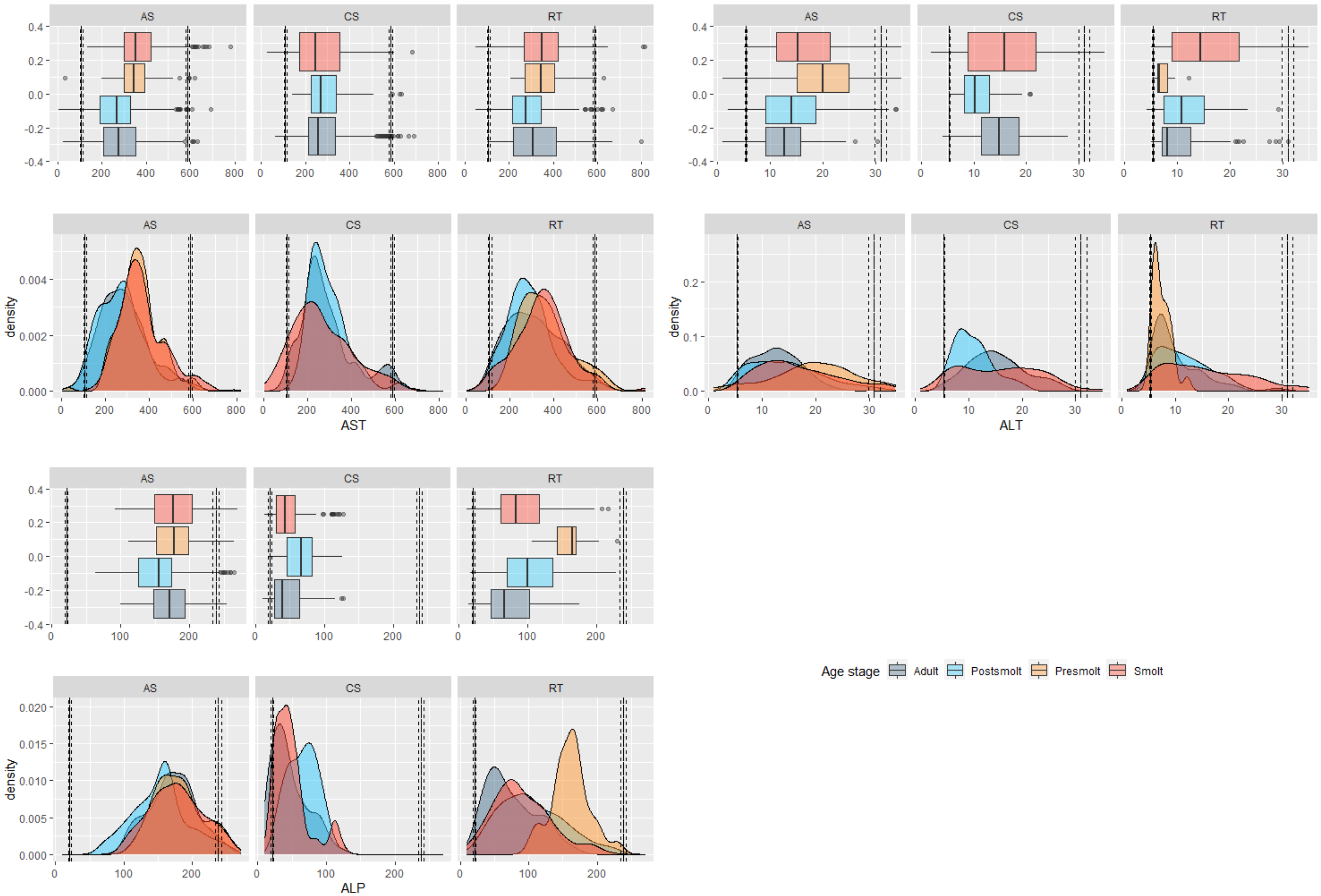

AS
CS
RT
density
AST
ALT
ALP
Age stage
Adult
Postsmolt
Presmolt
Smolt

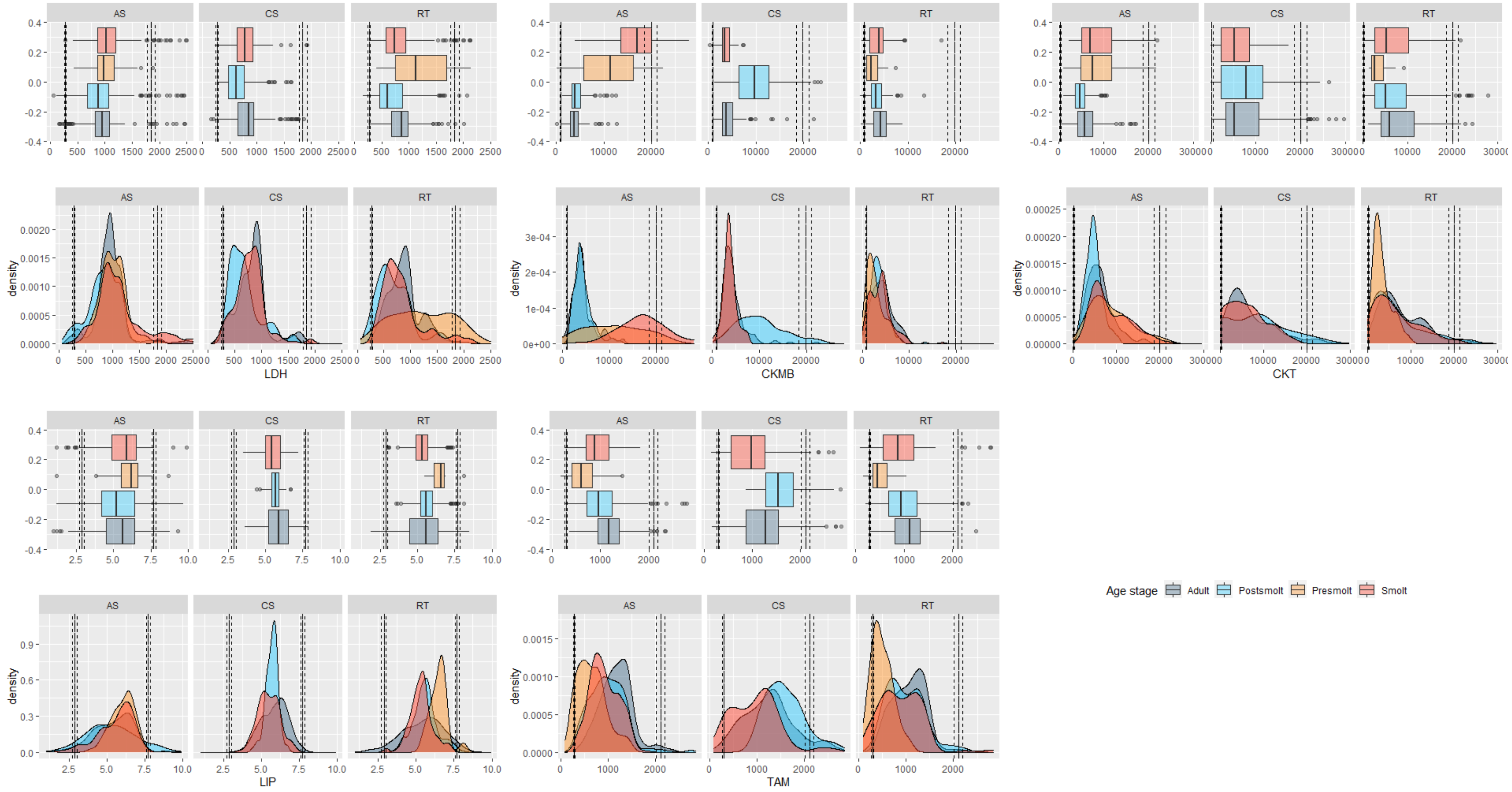

Figure S10. Histograms represent the count of plasma enzymes biomarkers observations and density plots show the distribution of data by estimating kernel density among salmonid species and age ranges. Box plots show the overall IRs (black lines) for plasma enzymes biomarkers. Dashed black lines show CIs for both the maximum and minimum range of RIs. AS: Atlantic salmon; CS: Coho salmon; RT: Rainbow trout.

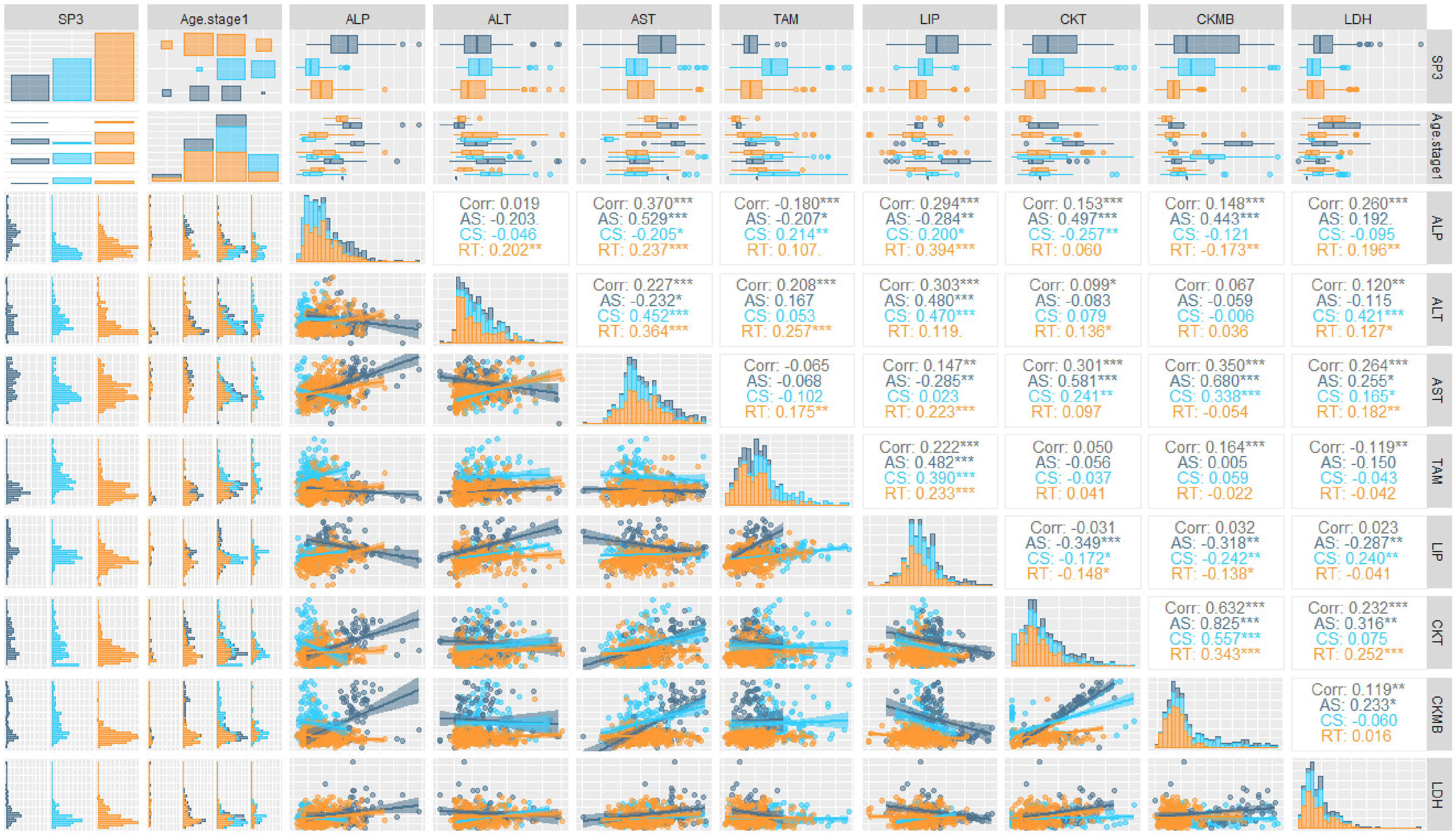

Figure S11. Correlogram between the different plasma enzymes biomarkers according to salmonid species. Pearson correlation coefficient (r) and p-value (p) between ALP, ALT, AST, TAM, LIP, CKT, CKMB, and LDH in Atlantic salmon (AS), coho salmon (CS) and rainbow trout (RT) (*p < 0.05, **p < 0.01, ***p < 0.001). SP: Specie.

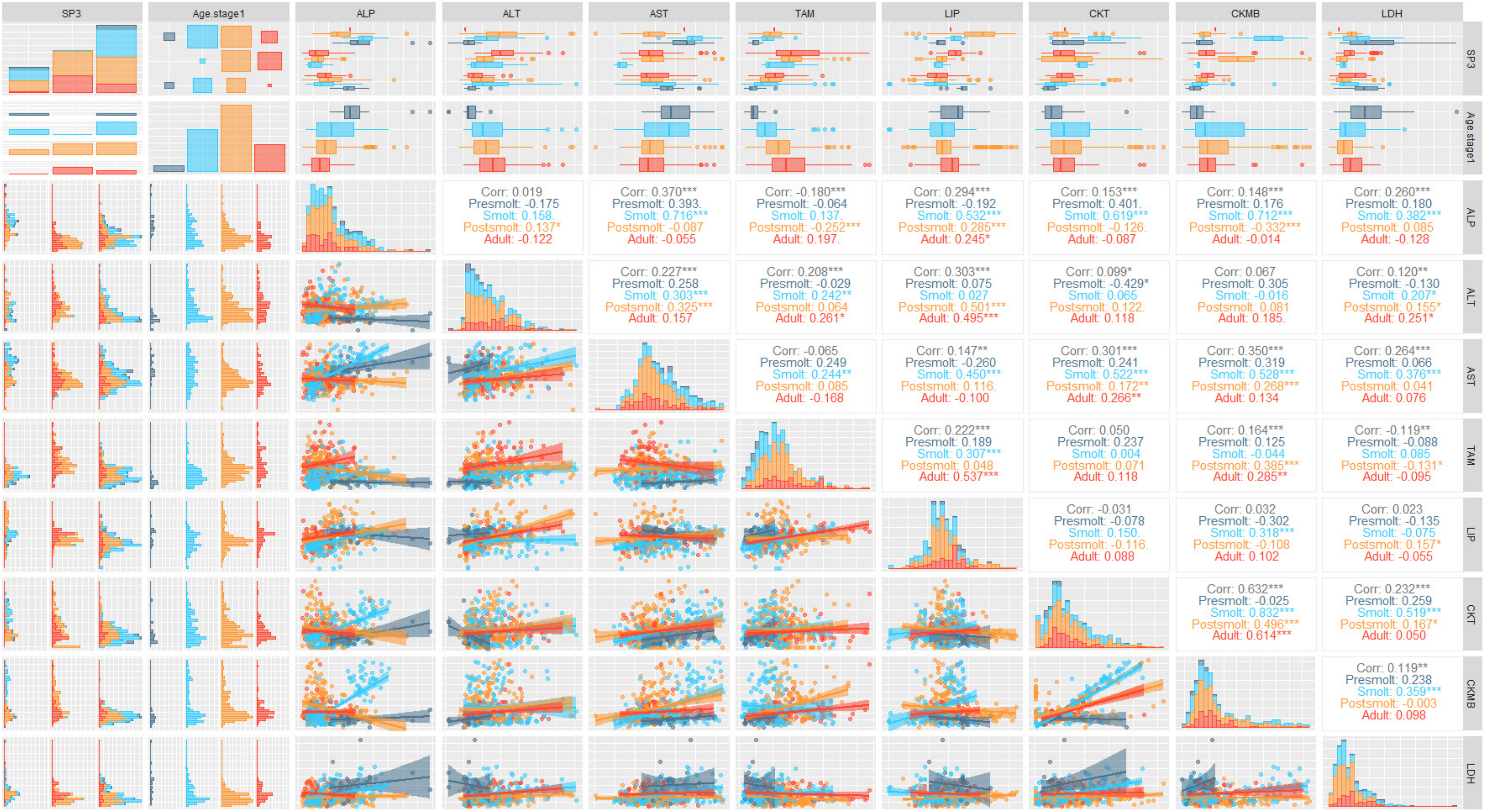

Figure S12. Correlogram between the different plasma enzymes biomarkers according to age ranges. Pearson correlation coefficient (r) and p-value (p) between ALP, ALT, AST, TAM, LIP, CKT, CKMB, and LDH in presmolt, smolt, postsmolt and adult from Atlantic salmon (AS), coho salmon (CS) and rainbow trout (RT) (*p < 0.05, **p < 0.01, ***p < 0.001). SP: Specie.

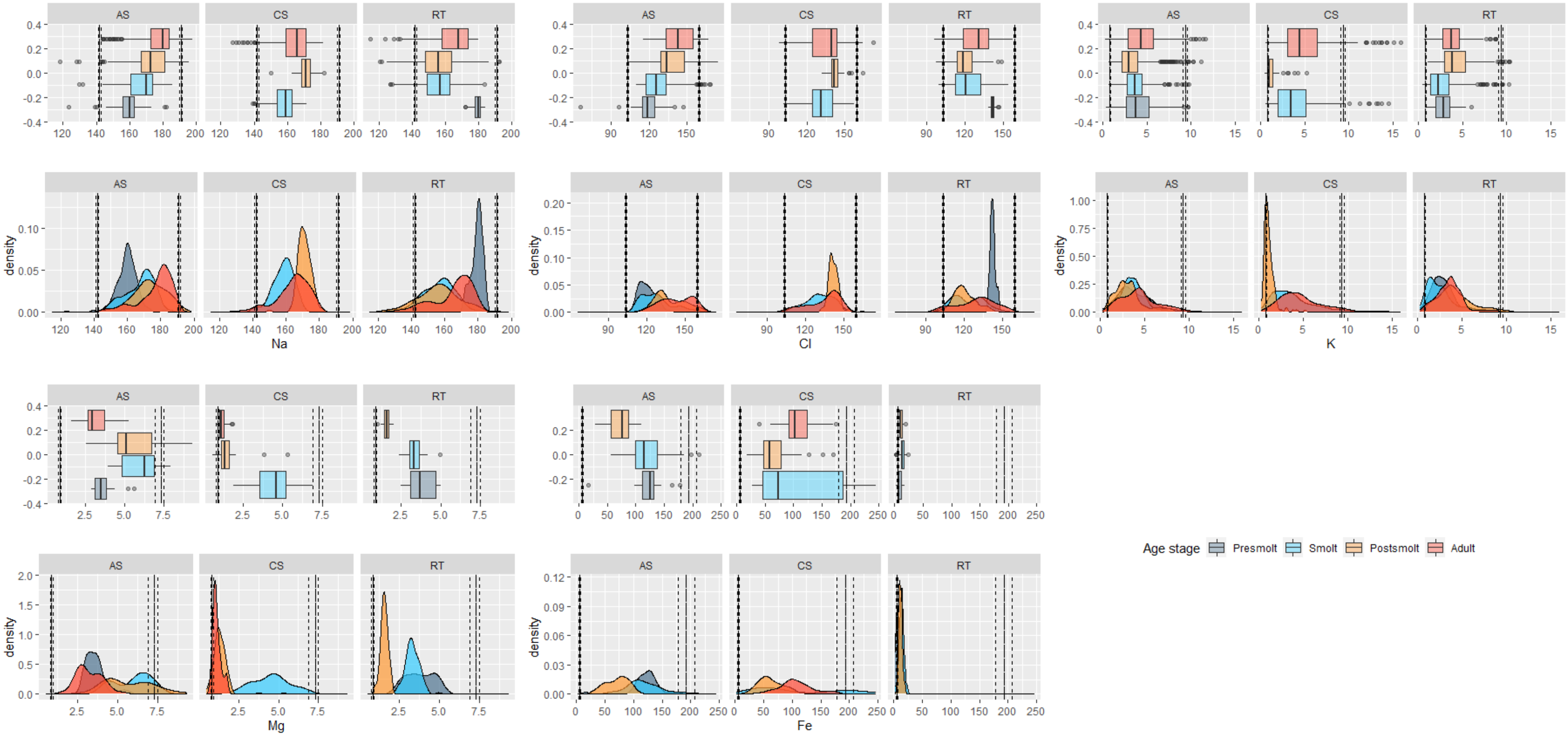

AS
CS
RT
density
Na
Cl
K
Mg
Fe
Age stage
Presmolt
Smolt
Postsmolt
Adult

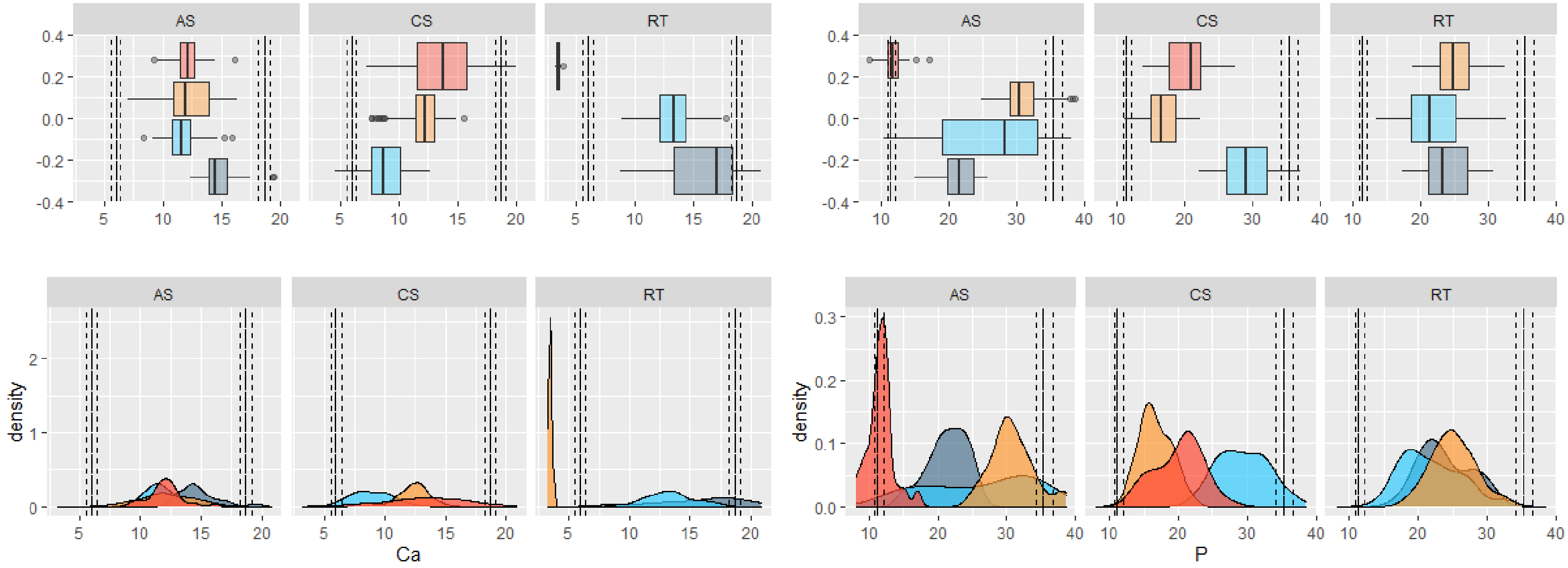

Figure S13. Histograms represent the count of plasma electrolytes and minerals biomarkers observations and density plots show the distribution of data by estimating kernel density among salmonid species and age ranges. Box plots show the overall IRs (black lines) for plasma electrolytes and minerals biomarkers. Dashed black lines show CIs for both the maximum and minimum range of RIs. AS: Atlantic salmon; CS: Coho salmon; RT: Rainbow trout.

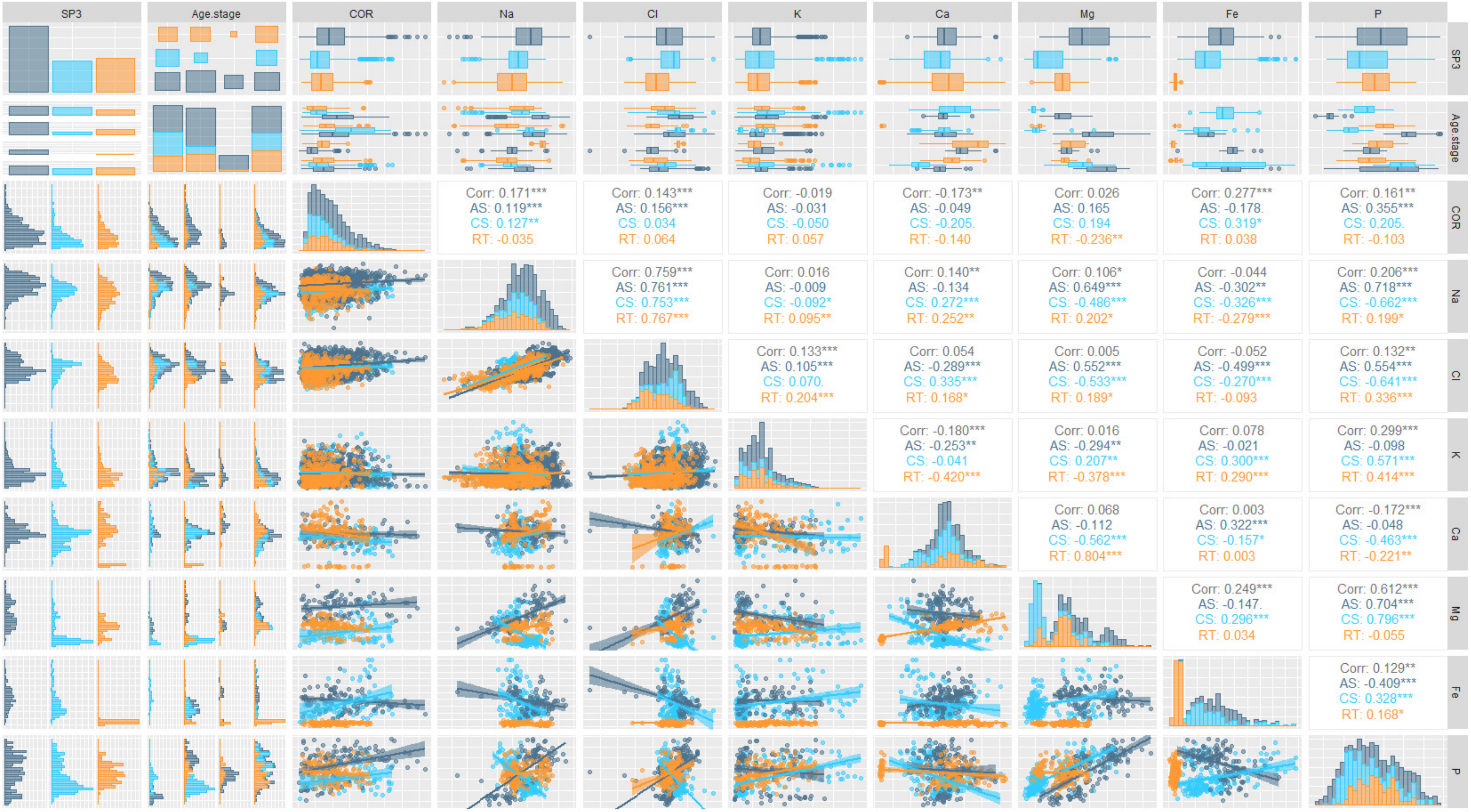

Figure S14. Correlogram between the different plasma electrolytes and minerals biomarkers according to salmonid species. Pearson correlation coefficient (r) and p-value (p) between Na, K, Cl, Ca, Mg, Fe, and P in Atlantic salmon (AS), coho salmon (CS) and rainbow trout (RT) (*p < 0.05, **p < 0.01, ***p < 0.001). SP: Specie.

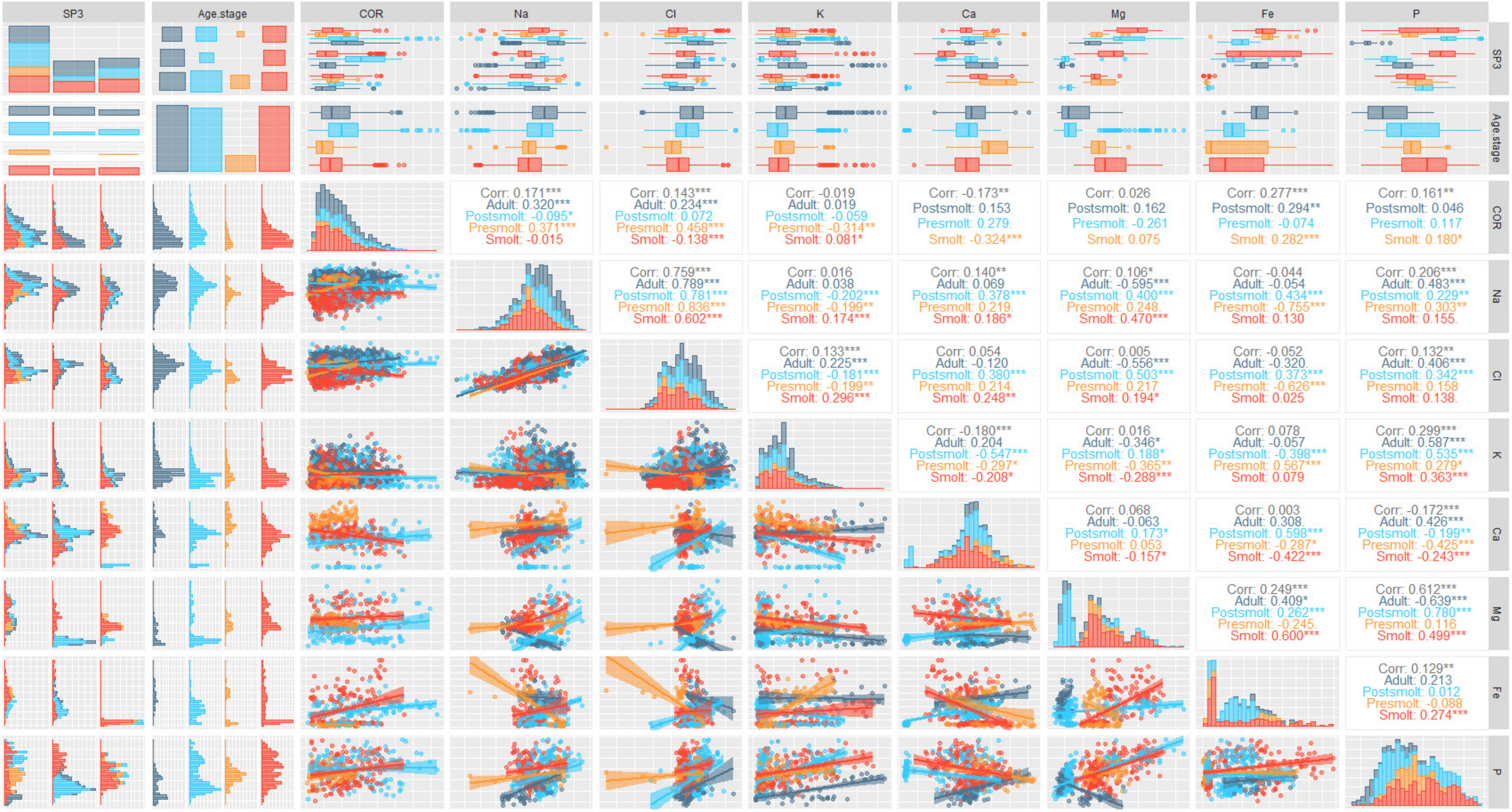

Figure S15. Correlogram between the different plasma electrolytes and minerals biomarkers according to age ranges. Pearson correlation coefficient (r) and p-value (p) between Na, K, Cl, Ca, Mg, Fe, and P in presmolt, smolt, postsmolt and adult from Atlantic salmon (AS), coho salmon (CS) and rainbow trout (RT) (*p < 0.05, **p < 0.01, ***p < 0.001). SP: Specie.

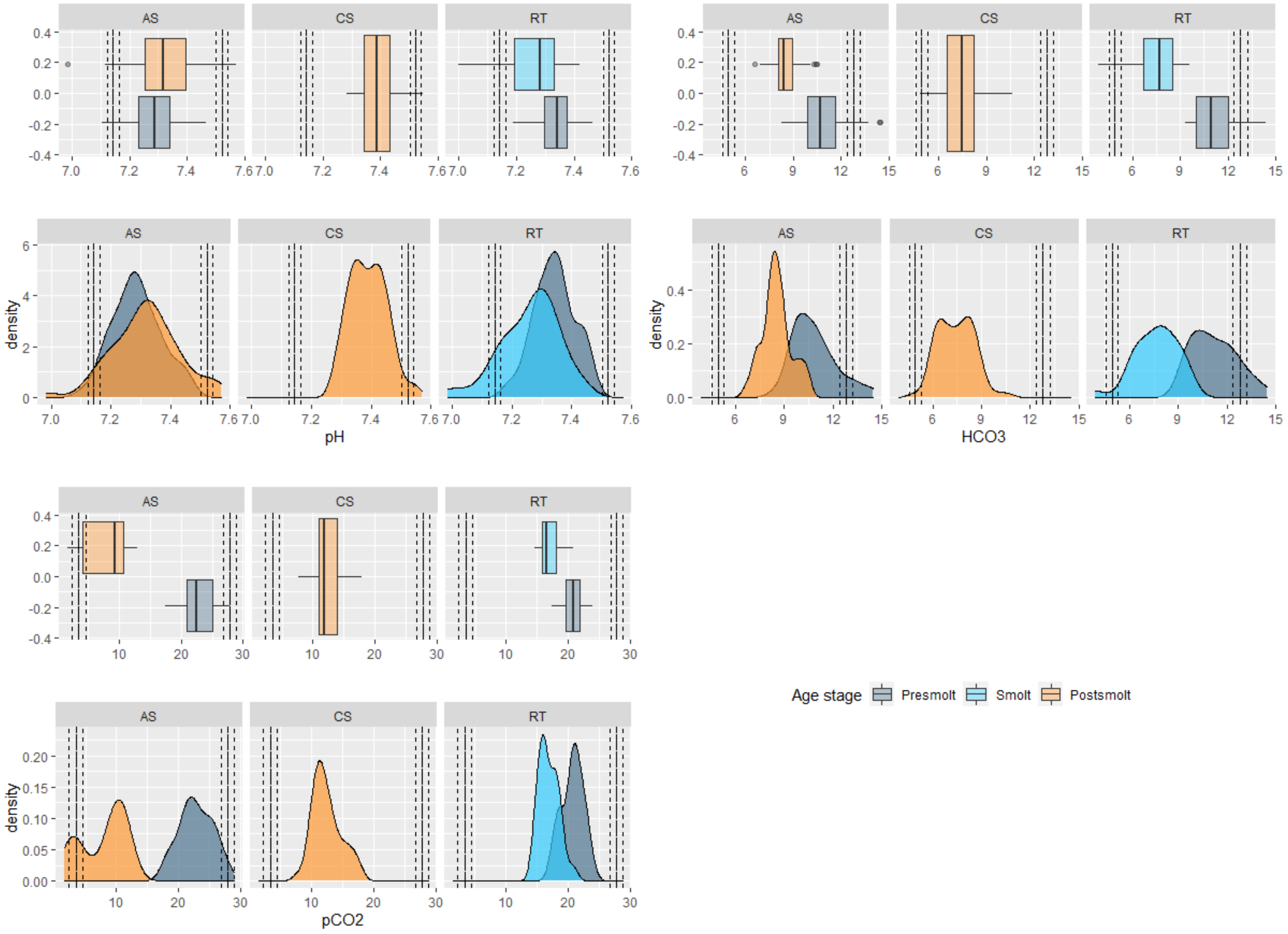

Figure S16. Histograms represent the count of blood gases observations and density plots show the distribution of data by estimating kernel density among salmonid species and age ranges. Box plots show the overall IRs (black lines) for blood gases. Dashed black lines show CIs for both the maximum and minimum range of RIs. AS: Atlantic salmon; CS: Coho salmon; RT: Rainbow trout.

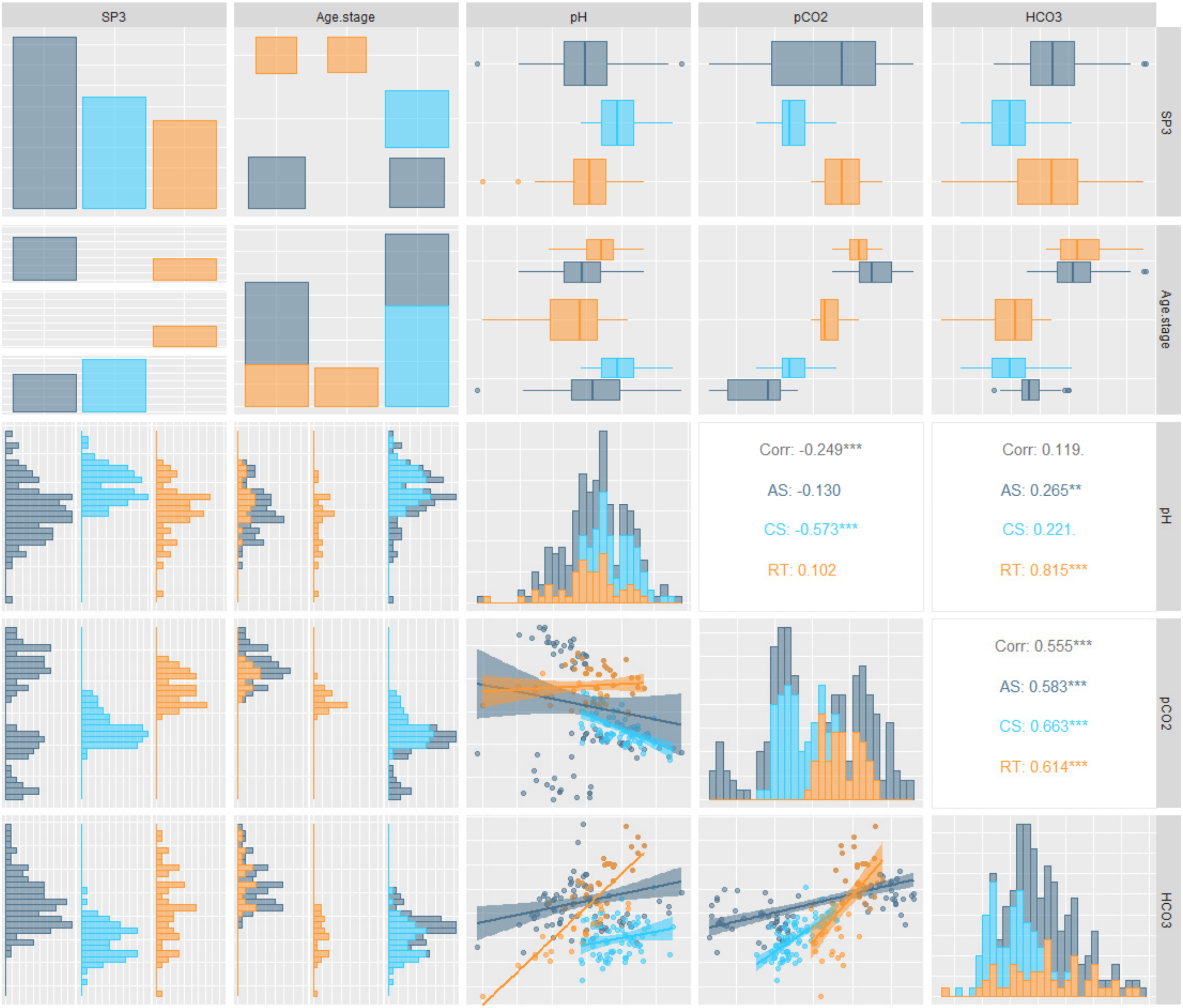

Figure S17. Correlogram between the different blood gases according to salmonid species. Pearson correlation coefficient (r) and p-value (p) between HCO_3, pCO_2, and pH in Atlantic salmon (AS), coho salmon (CS) and rainbow trout (RT) (*p < 0.05, **p < 0.01, ***p < 0.001). SP: Specie.

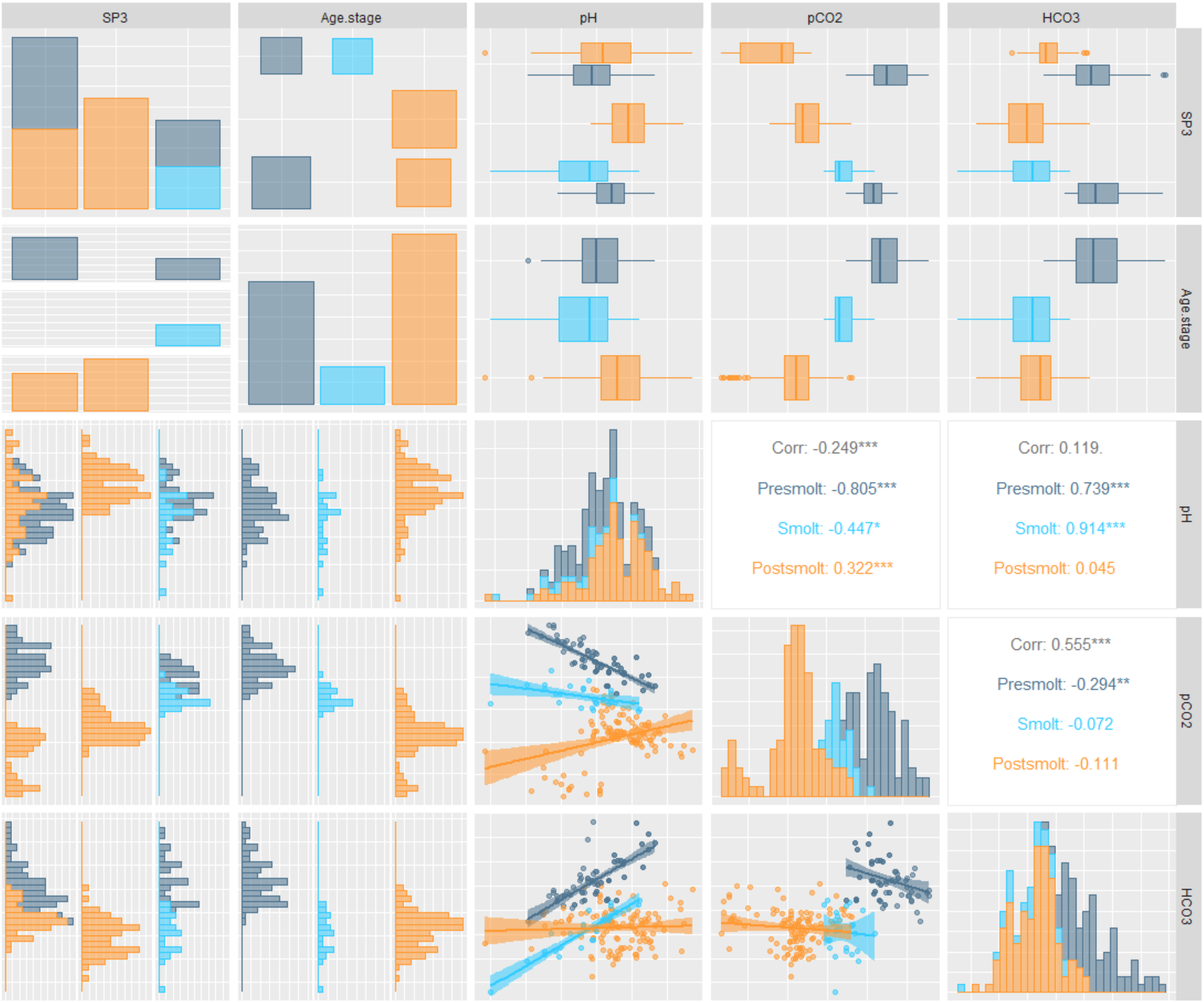

Figure S18. Correlogram between the different blood gases according to age ranges. Pearson correlation coefficient (r) and p-value (p) between HCO_3, pCO_2, and pH in presmolt, smolt, postsmolt and adult from Atlantic salmon (AS), coho salmon (CS) and rainbow trout (RT) (*$p < 0.05$, **$p < 0.01$, ***$p < 0.001$). SP: Specie.
